# MDMA-Induced Dissociative State not Mediated by the 5-HT_2A_ Receptor

**DOI:** 10.3389/fphar.2017.00455

**Published:** 2017-07-11

**Authors:** Drew J. Puxty, Johannes G. Ramaekers, Rafael de la Torre, Magí Farré, Neus Pizarro, Mitona Pujadas, Kim P. C. Kuypers

**Affiliations:** ^1^Department of Neuropsychology and Psychopharmacology, Faculty of Psychology and Neuroscience, Maastricht University Maastricht, Netherlands; ^2^Integrative Pharmacology and Neurosciences Systems Research Group, Institut Hospital del Mar d’Investigacions Mèdiques Barcelona, Spain; ^3^Spanish Biomedical Research Centre in Physiopathology of Obesity and Nutrition Santiago de Compostela, Spain; ^4^Facultat de Ciencies de la Salut i de la Vida, Universitat Pompeu Fabra Barcelona, Spain; ^5^Department of Pharmacology, Therapeutic and Toxicology, Universitat Autonoma de Barcelona Barcelona, Spain; ^6^Hospital Universitari Germans Trias i Pujol, Clinical Pharmacology Badalona, Spain

**Keywords:** MDMA, dissociative state, cortisol, heart rate, 5-HT_2_ receptor, MDMA concentration, depersonalization, derealization

## Abstract

Previous research has shown that a single dose of MDMA induce a dissociative state, by elevating feelings of depersonalization and derealization. Typically, it is assumed that action on the 5-HT_2A_ receptor is the mechanism underlying these psychedelic experiences. In addition, other studies have shown associations between dissociative states and biological parameters (heart rate, cortisol), which are elevated by MDMA. In order to investigate the role of the 5-HT_2_ receptor in the MDMA-induced dissociative state and the association with biological parameters, a placebo-controlled within-subject study was conducted including a single oral dose of MDMA (75 mg), combined with placebo or a single oral dose of the 5-HT_2_ receptor blocker ketanserin (40 mg). Twenty healthy recreational MDMA users filled out a dissociative states scale (CADSS) 90 min after treatments, which was preceded and followed by assessment of a number of biological parameters (cortisol levels, heart rate, MDMA blood concentrations). Findings showed that MDMA induced a dissociative state but this effect was not counteracted by pre-treatment with ketanserin. Heart rate was the only biological parameter that correlated with the MDMA-induced dissociative state, but an absence of correlation between these measures when participants were pretreated with ketanserin suggests an absence of directional effects of heart rate on dissociative state. It is suggested that the 5-HT_2_ receptor does not mediate the dissociative effects caused by a single dose of MDMA. Further research is needed to determine the exact neurobiology underlying this effect and whether these effects contribute to the therapeutic potential of MDMA.

## Introduction

Classical psychedelics like lysergic acid diethylamide (LSD), *N,N*-Dimethyltryptamine (DMT) and psilocybin are known for their mind-altering and dissociative states, as well as the spiritual or mystical-like experience often reported by users (Goodman, 2002; Trichter et al., 2009; Griffiths et al., 2011). These dissociative experiences, characterized by a disruption of cognitive and motor processes, can be viewed as a continuum ranging from excessive daydreaming and memory problems to more severe forms of depersonalization or derealization disorders (Association, 2013). Previously, it has been shown that a single dose of MDMA can also acutely induce a dissociative state, as measured with the Altered States of Consciousness Scale and the Clinician-Administered Dissociative State Scale (CADSS) (Vollenweider et al., 1998; van Heugten-Van der Kloet et al., 2015). It was found to exceed the non-psychotic state of schizophrenic patients, yet to be milder than the dissociative state induced by a typical psychedelic (psilocybin) or a dissociative (ketamine), and to be experienced as non-problematic by the psychedelic user (Vollenweider et al., 1998; Jansen, 2000; van Heugten-Van der Kloet et al., 2015). This effect has been shown to be dose-dependent, i.e., whereas low doses of MDMA (25–50 mg) did not induce a dissociative state, a higher dose (100 mg) did, 90 min after administration (van Heugten-Van der Kloet et al., 2015).

It is generally assumed that the hallucinogenic actions of classical psychedelics arise from their action on the serotonin 2A (5-HT_2A_) receptor (Glennon et al., 1984; Vollenweider and Kometer, 2010). Likewise, studies have demonstrated that 5-HT_2A_ receptors mediate MDMA-induced alterations in mood and perception (Liechti et al., 2000; van Wel et al., 2012). Specifically, pre-treatment with the 5-HT_2_ antagonist ketanserin selectively reduced MDMA-induced perceptual changes, emotional excitation, and alterations in positive affect (Liechti et al., 2000, 2001; Liechti and Vollenweider, 2001; van Wel et al., 2012). Based on this it was hypothesized that the 5-HT_2_ receptor could play a role in the MDMA-induced dissociative symptoms.

In addition, studies have demonstrated relationships between specific biological parameters like cortisol levels and heart rate, and state and/or trait dissociation in healthy and patient populations (Giesbrecht et al., 2007; Simeon et al., 2007). Previous studies have for example suggested a blunting of the autonomic responses, i.e., heart rate, skin conductance, and (nor)epinephrine levels, to stressful traumatic stimuli in acute dissociative states (Griffin et al., 1997; Delahanty et al., 2003). One study even showed an inverse relationship between cortisol stress reactivity and dissociation in dissociative disorder patients and post-traumatic stress disorder patients (Simeon et al., 2007). In healthy participants the cortisol response to a psychological stressor was shown to correlate positively with trait dissociation as measured with the depersonalization-derealization subscale of the Dissociative Experiences Scale (DES). Participants with the highest scores on dissociation also had the largest cortisol response to a stressor (Giesbrecht et al., 2007). In all these studies the stressor was psychological by nature, i.e., real-life events or stimuli, or laboratory procedures, causing the exposed person to experience psychological stress. It is also known that psychedelics, dissociatives, and MDMA produce a robust acute increase the body’s stress system, e.g., causing an elevation in cortisol levels and in cardiovascular parameters, making those substances to be categorized as ‘biological’ stressors (White and Ryan, 1996; Harris et al., 2002; Hasler et al., 2004; Parrott, 2009; Kuypers et al., 2013; van Heugten-Van der Kloet et al., 2015). Given this information, it would be relevant to study the association between these biological correlates of stress and MDMA-induced dissociation. In addition, since previous studies have shown MDMA concentrations in blood to correlate positively with the MDMA-induced changes in behavioral measures (e.g., emotional empathy and prospective memory) (Ramaekers et al., 2009; Kuypers et al., 2017), it would be relevant to explore the relation between MDMA concentrations and the dissociative state.

In order to study the effects of the 5-HT_2_ receptor in the MDMA-induced dissociative state, and the relationship to cortisol levels, heart rate, and MDMA concentrations, a placebo-controlled experimental study was set up including pretreatment with ketanserin, a 5-HT_2A_ blocker, and treatment with a single dose of MDMA (75 mg). It was hypothesized that MDMA would induce a dissociative state and that ketanserin would counteract this MDMA effect.

## Materials and Methods

### Participants

Participants were 20 healthy (12 males and 8 females) recreational poly-drug users with a mean age of 21.2 years (*SD*: 2.6), who had previously used ecstasy/MDMA with an average of 16.8 times (*SD*: 23.2) during their lifetime. They were recruited through advertisements at Maastricht University, a website (digi-prik.nl) and by word-of-mouth.

### Design and Treatments

The study was conducted according to a two by two double-blind placebo-controlled within-subjects design. Pre-treatment consisted of ketanserin (40 mg), which represents a regular therapeutic dose that blocks 91% of 5-HT_2_ receptors (Brogden and Sorkin, 1990; Sharpley et al., 1994), or placebo; Treatment consisted of MDMA (75 mg) or placebo. Pre-treatment and treatment were administered orally in identically appearing capsules using a double-blind, placebo-controlled, double-dummy procedure. A double-dummy procedure was used to control for differences in T_max_ between both drugs. T_max_ of MDMA is 2 h (de la Torre et al., 2004), T_max_ of ketanserin is between 0.5 and 4 h (Reimann et al., 1983; Heykants et al., 1986; Persson et al., 1991). The timing and doses of the (pre-)treatment were based on similar research by the same group (van Wel et al., 2011, 2012) were it was shown -amongst other- that the MDMA-induced elevated mood state was blocked by ketanserin (50 mg) (van Wel et al., 2012).

A permit for obtaining, storing, and administering MDMA was obtained from the Dutch drug enforcement administration. Randomization of pre-treatment and treatment conditions was generated by means of a Latin Square, with each subject being assigned to a treatment sequence.

### Procedure

Prior to participation all participants were medically assessed by a physician, who examined general health (including an ECG) and took blood and urine samples for standard chemistry and hematology. In addition, they were familiarized with the procedures, tests and questionnaires on a training day, and a questionnaire assessing dissociative trait was administered, preceding actual test days. Participants were requested to abstain from any drug use 1 week before the medical examination until the last test day. They were asked not to use any caffeinated or alcoholic beverages 24 h before testing and to get a normal night’s sleep.

Participants were screened for recent drug consumption in urine (THC, opiates, cocaine, amphetamine/ecstasy, and benzodiazepines) and alcohol in breath upon arrival (9 AM) on test days. In addition, women were given a pregnancy test. When tests were negative, participants had breakfast and filled out a questionnaire assessing baseline dissociative symptoms (CADSS), and blood samples were collected. At 9:30 AM participants received pre-treatment followed half an hour later by treatment. Participants were then seated in a waiting room for 90 min after which a second blood sample was taken and the CADSS was filled out again. The test day ended with a measurement of cardiovascular parameters and a third blood sample, at 12:30 PM, 150 min after treatment, 180 min after pre-treatment.

The procedure entailed four test sessions on four separate days, with a minimum of 7 days between test days as washout period. Participants provided written informed consent to participate in this study and were paid upon completion of the testing periods for their participation. The study was performed in accordance with the Helsinki Declaration of 1975 and its amendments, and was approved by the Medical Ethics Committee of the Academic Hospital of Maastricht and Maastricht University.

### Dissociative Trait and State Measures

#### Dissociative Experiences Scale

The DES is widely acknowledged as a standard instrument for trait dissociation. It comprises 28 items that assess the frequency of various dissociative phenomena in daily life. Participants indicate on 100-mm visual analog scales (anchors: 0 = not at all; 100 = very much) the percentage of time that they have experienced phenomena like talking out loud to oneself when one is alone or not recognizing friends or family members. The scale consists of five subscales, i.e., ‘Amnestic,’ ‘Absorption/Imagination,’ ‘Depersonalization/Derealization,’ ‘Absorption/Changeability,’ ‘Taxon DES scale.’ The latter scale provides a clinical mean cut-off score (cut-off score ≥ 20) suggesting further clinical assessment (Thomson and Jaque, 2012). To obtain a mean DES score, scores are averaged across items. Higher scores indicate a higher frequency of dissociative symptoms reported by the participant (Merckelbach et al., 2002).

#### Clinician-Administered Dissociative States Scale

The CADSS is an instrument developed for the measurement of present-state dissociative symptoms. The scale consists of a 19 self-report items and 8 observation items. The intensity of each dissociative symptoms ranges from 0 (not present at all) to 4 (extremely present). Respondents were instructed to use their current state (up too last 3 h) as a point of reference when completing items. We only employed the self-report items (1–19), and by summing across items we calculated a total score (0–76) and three subscales of ‘depersonalization’ (0–20), ‘derealization’ (0–48), and ‘amnesia’ (0–4).

### Biological Correlates of Stress and Pharmacokinetics

Heart rate and blood pressure (diastolic and systolic) were assessed three times during a test day followed each time by blood drawing, i.e., at baseline, pre-test, and post-test. Blood samples were collected in order to determine, MDMA and ketanserin.

#### Cortisol Concentrations

A 2-ml sample for cortisol analysis was drawn and collected in EDTA tubes. Samples were immediately centrifuged at 3000 rpm for 10 min at 4°C. Plasma was removed and frozen at -20°C until analysis. Cortisol samples were analyzed with the DPC IMMULITE 1000^®^ chemoluminescence immunoassay analyser (Siemens Healthcare Diagnostics).

#### Pharmacokinetics

Samples were centrifuged immediately and resulting plasma was stored at *-*20°C until analysis. MDMA, were determined by gas-chromatography coupled to mass spectrometry using a method previously described by Pizarro et al. (2002). Ketanserin was determined by liquid chromatography coupled to mass spectrometry. Samples (200 μL of plasma) were purified with Ostro Pass-through Sample Preparation Plates (Waters, Beverly, MA, United States) and 600 μL of acetonitrile with 0.1% formic acid was used as the elution solvent. After mixing, vacuum was applied and the collected mixture was evaporated to dryness at 15 psi and 40°C. Extract was reconstituted with 100 μL of ammonium formate 0.02% at pH 5 and acetonitrile (50: 50 v/v). Quantification was performed in an HPLC system coupled to a triple-quadrupole (6410 Triple Quad LC-MS; Agilent) mass spectrometer with an electrospray interface. The chromatographic separation was done using a C18 column (Kinetex, 100 mm × 3 mm × 1.7 μm, Phenomenex, Torrance, CA, United States). The mobile phase was ammonium formate 0.02% at pH 5 and acetonitrile in an isocratic mode (50: 50 v/v) at a flow rate of 0.45 mL/min. All compounds were monitored in positive ionization using the multiple reaction mode Mass/charge (M + 1/z) values selected for identification of analytes were as follows: ketanserin 396→146, 189, 208 and pirenperone 394→119, 159, 187, fragmentor (F) 200 V, collision energy (CE).

### Statistical Analysis

Clinician-Administered Dissociative State Scale data entered a general linear model (GLM), repeated measures procedure (SPSS, version 24.0) with Pre-treatment (two levels: ketanserin, placebo) and Treatment (two levels: MDMA, placebo) as main within subject factors.

For the cardiovascular parameters and cortisol concentrations, baseline measures were collected. First, a GLM was conducted, including only baseline to test for baseline differences. In case there were no differences, another GLM was conducted, including only the second (pre-test) or third (post-test) measure.

To study whether ketanserin and MDMA plasma concentrations differed significantly between conditions in which ketanserin or MDMA were administered alone or together, separated by 30 min, paired sample *t*-tests were conducted.

Pearson’s correlations were calculated in order to explore potential relationships between cardiovascular parameters and cortisol concentrations, and between these measures and measures of dissociation (DES, CADSS) and between MDMA concentrations and measures of dissociative state (CADSS). Pearson’s correlations were only conducted on the second measurement, which coincided with the assessment of the dissociative state.

The alpha criterion level of statistical significance for all analyses was set at *p* = 0.05; partial eta^2^ (η^2^) is reported in case of significant effects to demonstrate the effect’s magnitude (0.01: small, 0.06: moderate; 0.14: large).

## Results

### Dissociative Trait and State Measures

#### Dissociative Experiences Scale

Participants had a mean (± SE) total DES score of 13.8 (2.8). This score is in the range previously reported in healthy participants (Merckelbach et al., 2002). Mean scores for the DES-T, Amnesia, Absorption/Imagination, Depersonalization/Derealization, and Absorption/Changeability were 5.6 (2.2), 10.3 (2.9), 17.6 (3.9), 2.7 (1.6), and 16.6 (3.6), respectively.

#### Clinician-Administrated Dissociative States Scale

**FIGURE 1:**
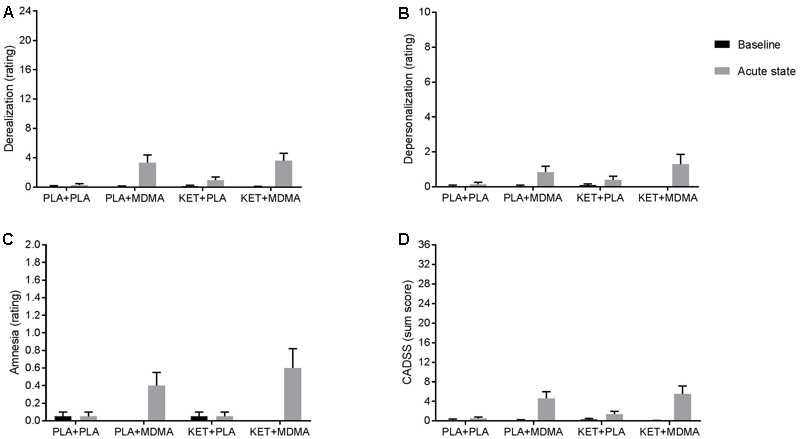
Mean (± SE) ratings on the subscales **(A)** Derealization, **(B)** Depersonalization, **(C)** Amnesia of the CADSS and the sum score **(D)** of the scales, during baseline and 120 and 90 min after pre-treatment and treatment respectively.

Analyses revealed a main effect of Treatment (MDMA) on the three CADSS subscales, i.e., Depersonalization, Derealization, and Amnesia (*F*_1,19_ = 11.62, *p* = 0.003; η^2^ = 0.38). MDMA induced an increase in the ratings on these scales compared to placebo. There was a main effect of Scales (*F*_2,38_ = 10.02, *p* < 0.001; η^2^ = 0.34) indicating significant differences between Derealization and both Depersonalization and Amnesia. The ratings on the latter scales were lower compared to the former scale. There was also a Treatment by Scale interaction (*F*_2,38_ = 10.97, *p* < 0.001; η^2^ = 0.37) indicating that the influence of MDMA on the Derealization rating was more pronounced than on the ratings of Depersonalization and Amnesia. There was no significant Pretreatment (ketanserin) by Treatment interaction effect, i.e., ketanserin did not change the effects of MDMA on dissociative experiences (**Figure 1**). There were no differences on baseline CADSS ratings between conditions.

### Biological Correlates of Stress and Pharmacokinetics

#### Cardiovascular Parameters

Baseline cardiovascular parameters did not reveal statistically significant differences between conditions. Mean (± SE) blood pressure (BP) and heart rate (HR) values were: 123.4 ± 1.8 mmHg for systolic BP, 71.6 ± 1.4 mmHg for diastolic BP, and 74.3 ± 1.5 bpm for HR.

Analysis including the second (i.e., pre-test) measurement revealed a main effect of Treatment on blood pressure and heart rate. Under influence of MDMA blood pressure and heart rate were elevated compared to placebo; respective differences were 12.5 mmHg (systolic BP), 6.2 mmHg (diastolic BP), and 10.4 bpm (HR).

Analysis including the third (i.e., post-test) measurement revealed main effects of Pre-treatment and Treatment on blood pressure and heart rate and an interaction effect on heart rate. The Treatment effect indicated an elevating influence of MDMA on blood pressure and heart rate; differences from placebo were respectively 10.9 mmHg (systolic BP), 8.2 mmHg (diastolic BP), and 13.2 bpm (HR). The Pre-treatment effect pointed to a reducing influence of ketanserin on blood pressure and heart rate; differences from placebo were respectively 7.8 mmHg (systolic BP), 6.1 mmHg (diastolic BP), and 3.4 bmp (HR). The Pre-treatment by Treatment interaction effect indicated that ketanserin partially counteracted the MDMA-induced increase in heart rate, i.e., heart rate was still higher in the combined condition but was lower than the MDMA only condition and still higher compared to placebo (**Table 1**).

**Table 1:** Mean (± SE) and general linear model (GLM) outcomes of the physiological measures, heart rate (HR) and blood pressure (BP); M = measurement; 1 = baseline; 2 = before cognitive tests (peak drug); 3 = after cognitive tests (end of test day); PT = pre-treatment; T = treatment; SBP = systolic blood pressure; DBP = diastolic blood pressure.

						GLM					
		Mean (± SE) conditions (PT+T)				PT		T		PT^∗^T	
	*M*	Placebo + Placebo	Placebo + MDMA	Ketanserin + Placebo	Ketanserin + MDMA	*F*	*p*	*F*	*p*	*F*	*p*
SBP	1	122.2 (3.3)	123.4 (3.4)	126.3 (4.1)	121.6 (4.0)	0.14	–	0.43	–	1.52	–
	2	120.8 (3.0)	136.2 (4.4)	122.6 (3.6)	132.3 (4.1)	0.15	–	27.39	0.00	0.80	–
	3	120.6 (3.3)	133.5 (2.6)	114.7 (3.3)	123.7 (3.7)	14.83	0.001	17.64	0.00	1.36	–
DBP	1	70.7 (2.2)	70.7 (2.0)	74.5 (3.8)	70.5 (2.8)	0.43	–	0.51	–	1.57	–
	2	69.9 (1.7)	75.5 (2.3)	70.7 (2.7)	77.4 (2.7)	0.44	–	12.60	0.002	0.07	–
	3	71.5 (1.8)	79.9 (2.5)	65.5 (1.7)	73.6 (2.5)	16.34	0.001	16.04	0.001	0.01	–
Heart rate	1	72.3 (2.6)	74.7 (3.4)	76.3 (3.1)	74.0 (3.3)	0.65	–	0.001	–	1.90	–
	2	69.3 (3.2)	81.9 (3.7)	72.7 (2.3)	80.9 (4.1)	0.27	–	16.37	0.001	0.73	–
	3	62.6 (1.7)	80.7 (2.8)	64.1 (2.4)	72.4 (2.7)	5.04	0.04	71.72	0.001	8.95	0.008

#### Cortisol Concentrations

**FIGURE 2:**
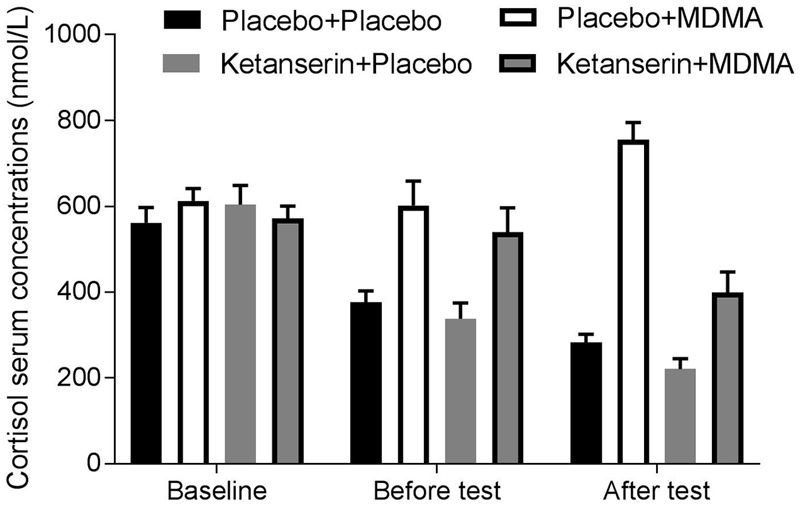
Mean (± SE) plasma concentrations of cortisol in the four treatment conditions, at baseline, before tests (i.e., 90 min after treatment, respectively 120 min after pre-treatment), and after tests (i.e., 150 min after treatment, respectively 180 min after pre-treatment).

Analyses revealed no differences in baseline cortisol serum concentrations (nmol/L). There was a main effect of Treatment on cortisol levels 90 (*F*_1,10_ = 9.70, *p* = 0.01; η^2^ = 0.49) and 150 (*F*_1,11_ = 65.67, *p* < 0.001; η^2^ = 0.86) minutes after administration. MDMA caused an elevation of cortisol concentrations compared to placebo, i.e., concentrations were 1.6 times higher after MDMA administration compared to placebo. Analyses revealed a main effect of Pre-treatment (*F*_1,11_ = 57.23, *p* < 0.001; η^2^ = 0.84) and a Pre-treatment by Treatment interaction effect (*F*_1,11_ = 40.49, *p* < 0.001; η^2^ = 0.79) on cortisol concentrations, 150 min after treatment and 180 min after pre-treatment administration respectively showing a counteraction of the MDMA-induced cortisol increase. When MDMA was combined with ketanserin, cortisol concentrations dropped with a factor of 1.9 compared to the MDMA only condition, but they were still 1.4 times higher compared to the placebo condition (**Figure 2**).

There was no effect of Pre-treatment or a Pre-treatment by Treatment interaction on cortisol levels 90 min after administration of treatment, respectively 120 min after pre-treatment.

#### Pharmacokinetics: MDMA and Ketanserin Concentrations

Paired sample *t*-tests showed that MDMA plasma concentrations (ng/mL) did not statistically differ between the MDMA alone condition [mean (± SE): 90′ post-MDMA: 134. 8 (16.6); 150′ post-MDMA: 186.0 (17.7)] and the condition where it was combined with ketanserin [mean (± SE): 90′ post-MDMA: 126.7 (15.1); 150′ post-MDMA: 182.9 (14.7)]. The same was shown for ketanserin plasma concentrations (ng/mL) that did not differ between the ketanserin alone condition [mean (± SE): 90′ post-MDMA: 54.9 (7.6); 150′ post-MDMA: 64.5 (6.0)] and the condition where it was combined with MDMA [mean (± SE): 90′ post-MDMA: 59.0 (8.8); 150′ post-MDMA: 61.5 (5.4)].

### Correlational Analyses

#### State and Trait Dissociative Experience

Trait dissociation as measured with the DES did not correlate with state dissociation as measured with the CADSS, i.e., Pearson’s correlations between DES-T and CADSS-Total ranged between -0.15 and 0.34, and correlations between DES-Total and CADSS-Total ranged between -0.07 and 0.30 for the four conditions.

#### Biological Correlates of the Dissociative State

##### Heart rate and cortisol levels

Analyses showed that heart rate and cortisol levels only correlated significantly in the MDMA-alone condition (*r*_15_ = 0.69; *p* = 0.005), 90 min after MDMA administration. Pearson’s correlations in the other conditions were not significant and ranged between -0.08 and 0.20.

Heart rate and cortisol levels did not correlate significantly 150 min after MDMA administration; correlations ranged between -0.28 and 0.44.

##### Cortisol levels and the dissociative state

Cortisol levels only correlated significantly with scores on the Amnesia subscale of the CADSS in the ketanserin only condition (*r*_19_ = 0.68; *p* = 0.001) and the placebo condition (*r*_18_ = 0.49; *p* = 0.04). Pearson’s correlations between cortisol levels and other subscales of the CADSS and the total score of the CADSS in the different conditions were not significant and ranged between -0.14 and 0.51.

##### Heart rate and the dissociative state

Heart rate correlated significantly with two subscales of the CADSS, i.e., Derealization (*r*_20_ = 0.62; *p* = 0.004) and Amnesia (*r*_20_ = 0.52; *p* = 0.02) and the total CADDS score (*r*_20_ = 0.60; *p* = 0.001) in the MDMA condition. The other Pearson’s correlations between heart rate and scales of the CADSS were not significant and ranged between -0.23 and 0.37.

##### MDMA concentrations and the dissociative state

Correlation analyses revealed only one significant association between MDMA concentrations in blood and dissociative state, i.e., MDMA concentrations in the combined ketanserin and MDMA condition correlated positively with the total score on the CADSS (*r*_18_ = 0.47; *p* = 0.05).

##### Dissociative traits, state, and the MDMA-related cortisol response

Since the cortisol response to a psychological stressor was previously shown to correlate positively with the depersonalization-derealization subscales of the DES (Giesbrecht et al., 2007) this association was also assessed in the present study. Cortisol response was defined as the percent change in the MDMA condition from placebo [((cortisol concentrations MDMA condition – cortisol concentrations Placebo condition)/cortisol concentrations Placebo condition)^∗^100]. The ‘cortisol response’ score was calculated separately for the MDMA condition in which ketanserin preceded MDMA treatment and in which placebo preceded MDMA treatment. Both scores were correlated with dissociative state and trait measures.

Trait depersonalization and derealization as measured with a subscale of the DES did not correlate significantly with the cortisol response in the MDMA conditions (*r*_14_ = -0.32 in the MDMA alone condition, and *r*_15_ = -0.17 in the combined ketanserin-MDMA condition).

State dissociation as measured with the subscales of the CADSS in the two MDMA conditions did not significantly correlate with the cortisol response in the MDMA conditions (range of r in MDMA alone condition = [-0.04 and 0.27]; ketanserin plus MDMA condition = [-0.22 and -0.13]).

## Discussion

The goal of this study was to investigate the role of the 5-HT_2A_ receptor in the MDMA-induced dissociative state and to examine whether biological measures such as heart rate, cortisol levels, and MDMA concentrations were correlated with this dissociative state. It was shown that a single dose of MDMA induced dissociative symptoms, i.e., 90 min after MDMA administration ratings of depersonalization, derealization, and amnesia were elevated. These effects were most pronounced for the derealization scale and they were not changed by ketanserin pre-treatment. MDMA caused an elevation of cortisol levels and heart rate 90 min after administration and these effects were partially counteracted by ketanserin, 150 min after MDMA administration. Correlational analyses between biological measures and the MDMA-induced dissociative state showed that heart rate was statistically significant related to these MDMA effects while MDMA and cortisol concentrations were not.

The effect of MDMA on dissociative symptoms replicates findings from previous research and extends these. While it was previously shown that lower single doses of MDMA (25–50 mg) did not induce a dissociative state and a higher dose (100 mg) did (van Heugten-Van der Kloet et al., 2015), it is now demonstrated that 75 mg of MDMA also induces a dissociative state. In line with previous research, ratings of derealization were elevated after MDMA administration (van Heugten-Van der Kloet et al., 2015), but in addition, 75 mg of MDMA also increased ratings of Depersonalization and Amnesia. The absence of an interaction between ketanserin and MDMA suggests a lack of involvement of the 5-HT_2A_ receptors in the MDMA-induced dissociative state effects. This notion is supported by the finding that MDMA-induced increases in ‘Oceanic Boundlessness,’ a subscale of the Altered State of Consciousness measuring derealization and depersonalization were not reduced by pre-treatment with ketanserin (50 mg) (Liechti and Vollenweider, 2001). Our biological measures show, however, that 60 min after CADSS completion, i.e., 150 min after MDMA administration, respectively 180 min after ketanserin administration, MDMA-induced elevations in cardiovascular parameters and cortisol levels were attenuated or even counteracted by ketanserin. This suggests that we should have measured dissociative symptoms a third time, coinciding the biological change. Conversely, Liechti and Vollenweider (2001) showing the same pattern on biological parameters did not demonstrate changes in subjective effects 120 min after MDMA administration, respectively 195 min after ketanserin administration. In addition, other stimulant drugs like cocaine and mephedrone are known to produce similar biological states without inducing dissociative states (Farré et al., 1997; Papaseit et al., 2016). Together these findings support dissociation between physiological and subjective effects, i.e., indicating that MDMA effects on physiological parameters are mediated -amongst other- by 5-HT_2A_ receptors and the dissociative state is not. However, future research should include multiple repetitions of the CADSS to exclude the possibility that dissociative effects are mediated by the 5-HT_2A_ receptor at later moments in time.

Additionally, if the 5-HT_2A_ receptor does not mediate this MDMA-induced dissociative state, the question is still open about which biological mechanism underlies this effect. In trauma-related disorders it has been shown that heightened glutamatergic neurotransmission occurs after stress exposure and this is related to the manifestation of dissociative states (Chambers et al., 1999). In addition, the increase in glutamate levels, caused by the NMDA receptor antagonist ketamine, has been shown to positively correlate with the degree of positive psychotic symptoms (Stone et al., 2012). From preclinical work it is known that MDMA causes an increase in glutamate levels, however, it is supposed to be caused indirectly via serotonergic stimulation of the 5-HT_2A/C_ receptor (Anneken and Gudelsky, 2012; Anneken et al., 2013). Experimental placebo-controlled human MDMA studies including proton magnetic resonance spectroscopy (^1^H-MRS) to assess brain glutamate concentrations together with measures of dissociative state would be a good starting point to confirm a relation between the MDMA-induced dissociative state and glutamate concentrations in the brain. These studies could be followed by dose-response blockade studies with MDMA and ketanserin in various doses to investigate the possibility whether ketanserin can block, at a different (higher) dose, the dissociative state and the potential glutamate increase by MDMA.

In the present study it was shown that the MDMA-induced dissociative state related positively to one biological measure, i.e., the effects on heart rate, but only when MDMA was administered alone, i.e., without ketanserin. This lack of correlation between the MDMA-induced dissociative state in the combined ketanserin-MDMA condition and heart rate reflects a discrepancy between effects of this pre-treatment-treatment combination on cardiovascular versus subjective measures. It also suggests that the heart rate *per se* is not directly related to experiencing a dissociative state, since normalizing the heart rate in the ketanserin-MDMA condition did not lead to a reduction of dissociative state. An interesting avenue for future research could be the inclusion of preselected high and low cortisol responders in relation to either a psychological or biological stressor conform previous research (Giesbrecht et al., 2007). In this way, the effect of stress reactivity after a stressor on dissociative state could be explored in a more rigorous manner since the current sample size did not allow separating groups based on cortisol response and studying this effect.

Since MDMA is currently being explored as adjunct to psychotherapy in post-traumatic stress disorder patients, it is relevant to know how MDMA could augment these therapeutic effects (Oehen et al., 2013; Mithoefer et al., 2013). Previously is has been suggested that depersonalization leads to ‘mind-emptiness,’ an indifference to pain, and an increased attentional state, associated with enhanced prefrontal cortex (PFC) activation and decreased anterior cingulate cortex activation (Sierra and Berrios, 1998). In addition it was proposed that derealization could lead to lowered emotionality, and lack of emotional coloring, linked to a PFC-driven inhibition of amygdala activity (Sierra and Berrios, 1998). Interestingly, studies have shown that MDMA leads to increases in blood flow in prefrontal areas and decreases in the amygdala and cingulate cortex (Gamma et al., 2000), or alternatively to a dampened amygdala reactivity (Bedi et al., 2009). These biological effects were accompanied by heightened mood and increased feelings of derealization (Gamma et al., 2000). Further research is needed to determine whether the MDMA-induced dissociative state is linked to this suggested indifference to pain, mind-emptiness, and lack of emotional coloring, all cognitive-emotional states that could help in processing traumatic experiences.

## Conclusion

It is suggested that the 5-HT_2_ receptor does not play a role in the MDMA-induced dissociative state. Heart rate correlates positively to this state but does not seem to be leading in this effect. Further research is needed to determine the exact neurobiology underlying this effect and whether these effects contribute to the therapeutic potential of MDMA.

## Author Contributions

KK, JR, RdlT, and MF have conceptualized the study design; KK has collected the data; KK, JR, RdlT, MF, NP, and MP have analyzed the data; KK, JR, RdlT, MF, and DP have written the manuscript.

## Conflict of Interest Statement

The authors declare that the research was conducted in the absence of any commercial or financial relationships that could be construed as a potential conflict of interest.
